# A high-density genetic map and QTL mapping of leaf traits and glucosinolates in *Barbarea vulgaris*

**DOI:** 10.1186/s12864-019-5769-z

**Published:** 2019-05-14

**Authors:** Tong-jin Liu, You-jun Zhang, Niels Agerbirk, Hai-ping Wang, Xiao-chun Wei, Jiang-ping Song, Hong-ju He, Xue-zhi Zhao, Xiao-hui Zhang, Xi-xiang Li

**Affiliations:** 1 Institute of Vegetables and Flowers, Chinese Academy of Agricultural Sciences; Key Laboratory of Biology and Genetic Improvement of Horticultural Crops, Ministry of Agriculture, Beijing, 100081 China; 2 Copenhagen Plant Science Center and Department of Plant and Environmental Sciences, University of Copenhagen, Thorvaldsensvej 40, 1871 Frederiksberg C, Denmark; 3 Henan Academy of Agricultural Sciences, Institute of Horticulture, Zhengzhou, 450002 China; 4 Vegetable Research Center, Beijing Academy of Agriculture and Forestry Sciences, Beijing, 100097 China

**Keywords:** *Barbarea vulgaris*, Restriction site-associated DNA sequencing (RAD-Seq), Genetic linkage map, QTL, Leaf traits, Glucosinolates

## Abstract

**Background:**

*Barbarea vulgaris* is a wild cruciferous plant and include two distinct types: the G- and P-types named after their glabrous and pubescent leaves, respectively. The types differ significantly in resistance to a range of insects and diseases as well as glucosinolates and other chemical defenses. A high-density linkage map was needed for further progress to be made in the molecular research of this plant.

**Results:**

We performed restriction site-associated DNA sequencing (RAD-Seq) on an F_2_ population generated from G- and P-type *B. vulgaris.* A total of 1545 SNP markers were mapped and ordered in eight linkage groups, which represents the highest density linkage map to date for the crucifer tribe Cardamineae. A total of 722 previously published genome contigs (50.2 Mb, 30% of the total length) can be anchored to this high density genetic map, an improvement compared to a previously published map (431 anchored contigs, 38.7 Mb, 23% of the assembly genome). Most of these (572 contigs, 31.2 Mb) were newly anchored to the map, representing a significant improvement. On the basis of the present high-density genetic map, 37 QTL were detected for eleven traits, each QTL explaining 2.9–71.3% of the phenotype variation. QTL of glucosinolates, leaf size and color traits were in most cases overlapping, possibly implying a functional connection.

**Conclusions:**

This high-density linkage map and the QTL obtained in this study will be useful for further understanding of the genetic of the *B. vulgaris* and molecular basis of these traits, many of which are shared in the related crop watercress.

**Electronic supplementary material:**

The online version of this article (10.1186/s12864-019-5769-z) contains supplementary material, which is available to authorized users.

## Background

*Barbarea vulgaris* (2n = 2x = 16) is a wild crucifer with an estimated genome size of 270 Mb [1]. It originated in the Mediterranean area and now occurs in temperate regions worldwide [2]. There are two morphologically, chemically and biologically distinct types of *B. vulgaris*: the G-type named after its glabrous leaves and the P-type named after its pubescent leaves [3–5]. The G-type is strongly resistant to two important agricultural pests, the diamondback moth (*Plutella xylostella*) and western flower thrips (*Frankliniella occidentalis*), while the P-type is completely susceptible to them [6–8]. A similar pattern was reported for a range of other herbivorous insects and mollusks [3, 9] and the powdery mildew fungus *Erysiphe cruciferarum* [8]. In contrast, the G-type was reported to be susceptible to the oomycete *Albugo candida* while the P-type was resistant to it [10]. The G- and P-types differ in the composition of glucosinolates, flavonoids and saponins, which are secondary metabolites known to play important roles in plant resistance to a series of biotic and abiotic stresses [11–15]. The G-type *B. vulgaris* is resistant to the diamondback moth and the flea beetle *Phyllotreta nemorum* due to its content of triterpenoid saponins, a unique feature among crucifers [6, 16–18], while the difference in *Albugo candida* resistance between G- and P-type plants could possibly be related to differences in glucosinolate composition and hydrolysis products [12] or glucosinolate derived phytoalexins [19, 20].

The above findings define *Barbarea vulgaris* as an important genetic resource for pest and disease resistance as well as genes responsible for glucosinolates and saponins. Glucosinolates are highly attractive for diamondback moth oviposition, while deterrence conferred by saponins kill the resulting larvae. Thus G-type *B. vulgaris* has a potential use in ‘dead-end trap cropping’ for diamondback moth management [21–23]. Another important goal of *B. vulgaris* research is to confer resistance traits (resistance to diseases and pests, cold tolerance, etc.) to cultivated crucifers. To achieve this goal, intertribal somatic hybridization between *Brassica napus* and *B. vulgaris* was tried but the hybrid plants could grow under in vitro conditions but not be recovered, revealing compatibility problems between the species [24]. Due to more recent progress in genetic transformation technology [25–27], identification of resistance genes in *B. vulgaris* can benefit from biotechnology-based breeding of cruciferous crops.

QTL (Quantitative Trait Locus) mapping is an effective approach widely used to identify genes determining a certain trait [28]. The creation of a genetic map using molecular markers is the first step in QTL mapping. Three previous reports have investigated the construction of the linkage maps of *B. vulgaris* and genetic dissection of its resistance to flea beetle larvae, using the same P × G-type derived F_2_ population. The first genetic linkage map included 100 AFLP (Amplified Fragment Length Polymorphism) and 31 SSR (Simple Sequence Repeat) markers with a total length of 889 cM and an average marker distance of 7.1 cM [14], which were distributed acroos eight linkage groups in agreement with the chromosome number [14, 29]. In the second genetic linkage map, more SSR markers were added to first linkage map resulting in a new linkage map. They were distributed on 17 linkage groups, which did not correspond to the chromosome number [30]. More recently, a high-density genetic map comprising 796 SNP markers on eight linkage groups was constructed using the same P × G-type derived F_2_ population by a genotype-by-sequencing approach [1]. This map was used to anchor 431 contigs (38.7 Mb, 23% of the assembly genome) in the *B. vulgaris* whole-genome sequencing project [1]. However, this map is still not sufficiently dense because of the limitation of the relatively small mapping population [31, 32], leading to reported difficulties in molecular studies of *B. vulgaris* defenses [1, 17, 30]. A higher density map would hence benefit *B. vulgaris* genome anchoring and fine mapping of QTL.

In this study, by application of Restriction site-associated DNA sequencing (RAD-Seq) strategies and expansion of the mapping population to 255 individuals, a new high-density linkage map with 1545 SNP markers was constructed. The genetic map allowed us to anchor 722 contigs from the sequenced G-type *B. vulgaris*. We further used this map to identify QTL underlying trichome density, maximum leaf length and width, color and glucosinolates of rosette leaves.

## Results

### RAD sequencing, SNPs detection and genotyping

In the present study, a total of 137.4 G (average 539.0 Mb per plant) raw data was generated by Illumina HiSeq platform sequencing from the F_2_ population and 134.5 G (527.6 Mb per plant on average) high-quality clean data was obtained after trimming adaptors and filtering out low quality reads. For the two parents, significantly higher depth sequencing was performed, generating 3.3 G and 3.5 G clean data for G-type and P-type plants, respectively. After removal of redundancy, 7,479,898 and 8,271,583 RAD-tags were identified from the G-type and P-type plants, respectively (Additional file 1: Figure S1a). The F_2_ individuals had an average of 1,277,043 tags (Additional file 1: Figure S1a). The sequencing depth of the RAD-tags was 43.93× for the G-type, 44.20× for the P-type, ranging from 7.07 to 12.53 with an average of 8.82× for F_2_ individuals (Additional file 1: Figure S1b).

The RAD-tags from G-type and P-type plants were assembled to 114,989 and 125,940 contigs with the total length of 37,549,170 and 39,800,608 bp, respectively (Table 1). The average contig length of G- and P-type was 326 and 316 bp with N50 sizes of 389 and 383 bp, respectively (Table 1). The assembled P-type contigs were used as reference for SNP discovery.

**Table 1:** Summary statistics of G- and P-type *Barbarea vulgaris* contigs assembly

Sample	Total contig base (bp)	Total contig number	Average contig length (bp)	N50 length (bp)	GC (%)
G	37,549,170	114,989	326	389	36.42
P	39,800,608	125,940	316	383	36.86

Between G-type and P-type *B. vulgaris*, a total of 80,928 SNPs were identified (~ 1 SNP/2 kb) (Table 2). Among these, 60,287 co-dominant “aa × bb” type SNPs were identified (Table 2). Subsequently, the polymorphic markers were detected in the F_2_ population, resulting in 8448 SNPs that were present in at least 216 plants (85% of the population). After segregation distortion filtering with *P*-value < 0.001, the remaining 2052 SNP markers were finally used for linkage map construction.

**Table 2:** Numbers of markers for four segregation types

Marker Type	Number
nn × np	7920
aa×bb	60,287
lm × ll	11,786
hk × hk	935
Total markers	80,928

“ll”, “aa”, homozygous genotype as in paternal; “lm”, heterozygous genotype as in maternal; “nn”, “bb”, homozygous genotype as in maternal; “np”, heterozygous genotype as in paternal; “hh”, “kk”, homozygous genotype in F_1_ progeny, and “hk” types represent heterozygous genotype as in paternal or maternal

### Linkage map construction

**Fig. 1:**
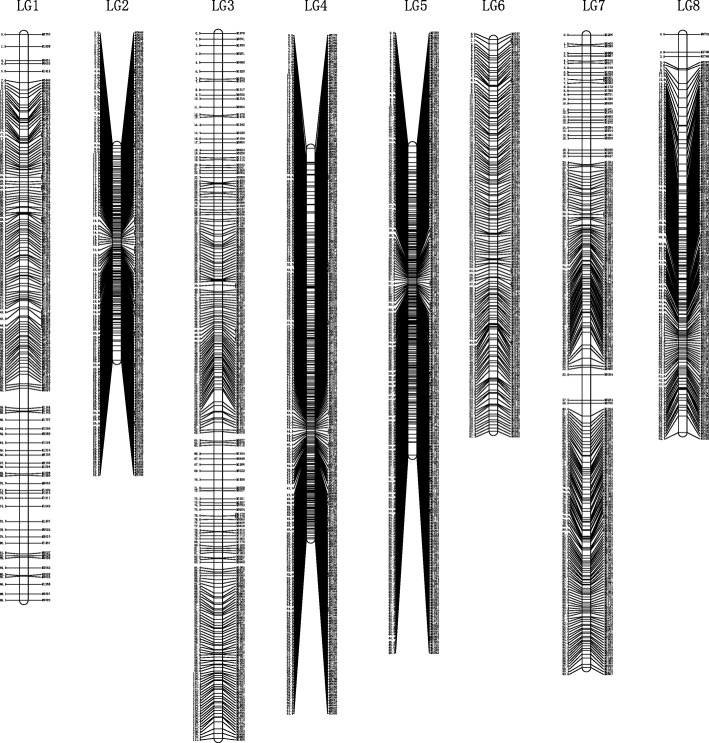
The high-density genetic map of *Barbarea vulgaris*. Map distances based on Kosambi map units (CM) are shown on the left margin and the marker names are shown on the right margin of each linkage group

Before construction of linkage maps, 286 similar SNP sites (similarity > 0.95) were excluded and the remaining markers were divided into eight groups using JoinMap 4.0. As a result, severely unlinked markers were excluded, and 1545 markers were mapped onto eight linkage groups (LGs) with a total length of 567.996 cM and an average distance of 0.381 cM between adjacent markers (Fig. 1, Table 3). On this map, LG3 (110.97 cM including 219 markers) and LG2 (33.804 cM including 168 markers) achieved the longest and shortest genetic distance. LG8 and LG4 had the highest and lowest marker number, 257 markers spanning 61.482 cM and 146 markers spanning 62.723 cM, respectively. The average gaps between adjacent markers of individual LGs varied from 0.202 to 0.582 cM (Table 3).

**Table 3:** Summary statistics for the *Barbarea vulgaris* genetic map

Linkage Group	Number of SNP	Length (cM)	Average Gap (cM)
LG1	154	89.082	0.582
LG2	168	33.804	0.202
LG3	219	110.970	0.509
LG4	257	61.482	0.240
LG5	235	48.659	0.208
LG6	153	61.716	0.406
LG7	213	99.560	0.470
LG8	146	62.723	0.433
Total	1545	567.996	0.381

### Anchoring the contigs

**Fig. 2:**
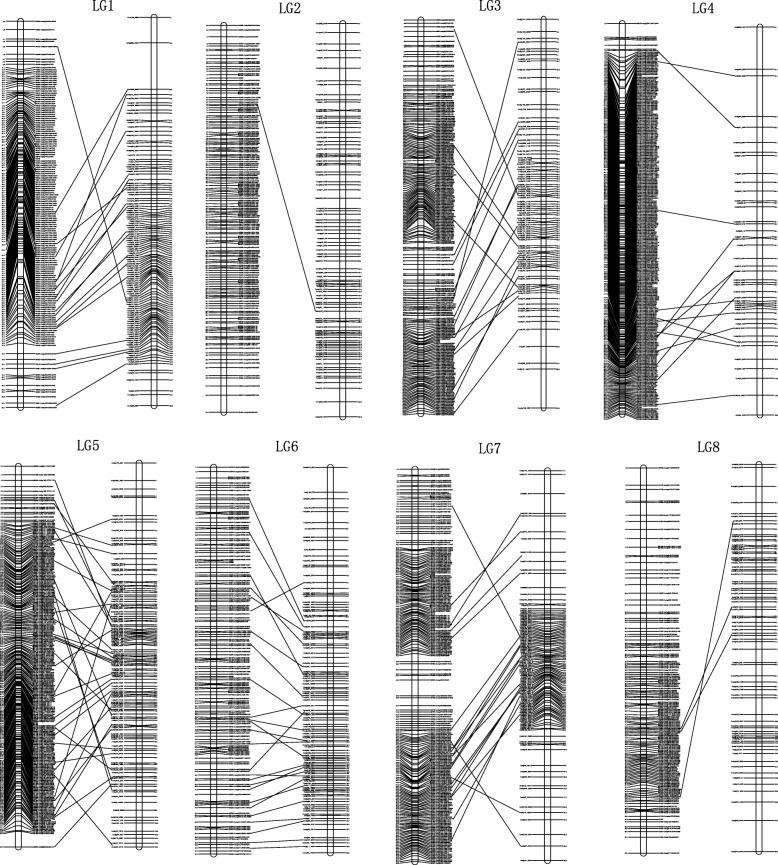
Anchoring of sequenced contigs of G-type *Barbarea vulgaris* to the eight linkage groups and correspondence with a previously published high density linkage map. The present and previously published linkage groups are shown to the left and right, respectively. Map distances based on Kosambi map units (CM) are shown on the left margin and marker names and anchored contigs are shown on the right margin of each linkage group for the present linkage maps, while map distances are shown on the right margin and anchored contigs are shown on the left margin for the previous linkage maps. Markers that harbor the same sequenced contig are connected by a straight line between the present and previous maps

The present linkage map was used to anchor contigs generated by a previous genome sequencing project [1]. The markers harboring RAD-Seq contigs of the genetic linkage map were aligned to the *B. vulgaris* genome contigs. As a result, excluding 16 SNP markers, 1, 529 SNP markers were mapped to a total of 722 contigs with a physical length of 50.2 Mb. Some contigs were anchored by more than one SNP marker (Fig. 2, Additional file 2: Table S1 and S2). Our map contains 150 contigs (with a total physical length of 19.0 Mb) which were also anchored on the published *B. vulgaris* genome [1]. Using these 150 shared contigs, the linkage groups were numbered in accordance with the previous one (Fig. 2). In addition, 572 contigs covering 31.2 Mb of physical length were newly anchored on the current map (Additional file 2: Table S2).

### QTL for trichomes, leaf size, color and glucosinolates

**Fig. 3:**
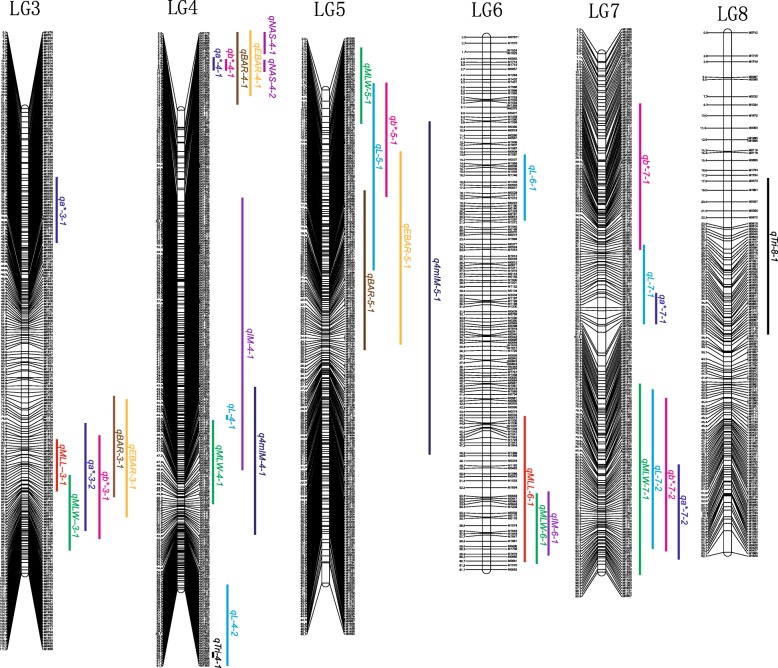
The QTL positions on the high density map of the F_2_ population. QTL names refer to Table 4

QTL analysis was performed for trichome density, leaf size, color and glucosinolates in the F_2_ population (Fig. 3 and Table 4). Two QTL associated with trichome density were identified on linkage groups (LG) 4 and 8, explaining 4.8 and 14.0% of the phenotypic variance, respectively. The additive effects of these QTL are negative values, in accordance with the fact that trichome density was contributed by P-type *B. vulgaris* (Table 4).

**Table 4:** QTL associated with trichome density, leaf size and color, and glucosinolates in *Barbarea vulgaris* rosette leaves

Trait	Linkage Group	QTL	LOD	PVE (%)	Intervals on maps (cM)	Additive effect
Trichome density	4	*qTri-4-1*	2.5	4.8	61.0–61.5	− 0.33
	8	*qTri-8-1*	5.8	14.0	17.4–37.6	−0.47
MLL	3	*qMLL-3-1*	3.9	5.9	79.6–89.9	−0.54
	6	*qMLL-6-1*	7.9	19.7	43.8–61.0	−0.86
MLW	3	*qMLW-3-1*	3.0	6.2	86.0–97.3	−0.60
	4	*qMLW-4-1*	3.1	8.7	45.2–50.6	−0.50
	5	*qMLW-5-1*	4.7	12.9	2.6–11.4	−0.63
	6	*qMLW-6-1*	4.9	7.8	52.7–61.1	−0.21
	7	*qMLW-7-1*	2.7	9.4	71.9–95.3	−0.61
L	4	*qL-4-1*	2.5	3.7	45.1–45.6	−0.44
	4	*qL-4-2*	3.5	4.5	55.6–61.5	−0.54
	5	*qL-5-1*	4.4	8.5	7.8–20.9	−0.65
	6	*qL-6-1*	3.8	7.5	13.5–21.2	−0.52
	7	*qL-7-1*	3.0	8.6	37.2–57.5	−0.48
	7	*qL-7-2*	3.0	6.9	72.9–92.3	−0.45
a*	3	qa*-3–1	3.4	6.4	34.6–42.9	−0.411
	3	qa*-3–2	8.5	17.2	76.5–94.5	0.60
	4	qa*-4–1	3.0	5.8	6.8–10.0	−0.37
	7	qa*-7–1	2.6	5.5	44.4–58.0	0.23
	7	qa*-7–2	5.3	7.9	83.0–90.7	0.53
b*	3	qb*-3–1	4.4	7.7	79.1–95.9	−0.86
	4	qb*-4–1	3.1	6.2	7.4–9.8	0.63
	5	qb*-5–1	4.0	7.7	7.8–16.4	−0.91
	7	qb*-7–1	4.7	7.4	18.7–38.2	−0.94
	7	qb*-7–2	6.3	8.0	73.8–92.7	−1.11
NAS	4	*qNAS-4-1*	6.5	42.3	0.20–5.9	2.71
	4	*qNAS-4-2*	4.9	19.3	7.2–10.0	1.98
BAR	3	*qBAR-3-1*	23.9	12.5	68.9–89.0	19.07
	4	*qBAR-4-1*	21.1	41.6	0.0–16.3	26.45
	5	*qBAR-5-1*	4.6	9.4	16.2–25.2	−9.2
EBAR	3	*qEBAR-3-1*	10.4	6.4	71.2–93.2	−10.84
	4	*qEBAR-4-1*	21.6	71.3	0.0–15.0	−19.04
	5	*qEBAR-5-1*	7.1	6.8	13.5–24.8	−7.04
IM	4	*qIM-4-1*	7.5	9.2	28.2–48.4	0.52
	6	*qIM-6-1*	5.4	16.9	52.8–60.1	0.39
4mIM	4	*q4mIM-4-1*	6.4	2.9	43.3–52.5	−0.11
	5	*q4mIM-5-1*	8.7	37.2	11.1–30.9	0.11

PVE, the percentage of variation explained;

Additive effect, positive additivity indicated that G-type *Barbarea vulgaris* carries the allele for an increase in the trait value, while negative additivity means that P-type plants carries the allele for an increase in the trait value;

MLL, maximum leaf length; MLW, maximum leaf width;

L, lightness; a*, redness and greenness and b*, yellowness and blueness;

NAS, 2-phenylethylglucosinolate (gluconasturtiin); BAR, (2*S*)-2-hydroxy-2-phenylethylglucosinolate (glucobarbarin); EBAR, (2*R*)-2-hydroxy-2-phenylethylglucosinolate (epiglucobarbarin); IM, 3-indolylmethylglucosinolate (glucobrassicin); 4mIM, 4-methoxy-3-indolylmethylglucosinolate (4-methoxyglucobrassicin)

QTL associated with maximum leaf length were identified on LG3 and LG6, explaining 5.9 and 19.7% of the phenotypic variance, respectively. QTL associated with maximum leaf width were identified on LG3, 4, 5, 6 and 7, explaining 6.2, 8.7, 12.9, 7.8 and 9.4% of the phenotypic variance. The QTL for leaf length and width on both LG3 and LG6 overlapped, while the additional QTL for leaf width did not reveal any relationship to leaf length.

The leaf color data were divided into three parameters, L (lightness), a* (redness and greenness) and b* (yellowness and blueness), for QTL analysis. Six, five and five QTL were identified to associate with L, a*, and b*, explaining 39.7, 42.8 and 37.0% of the total phenotypic variance of these traits, respectively. These QTL exhibited a complex organization, including both overlap and independent occurrence. Four out of five QTL for both a* or b* and three out of six QTL for L were overlapping with at least one other leaf color QTL. The QTL located at 79.1–95.6 cM on LG3 and at 6.8–10.0 cM on LG4 contributed to both a* and b*; a QTL located at 37.2–58.0 cM on LG7 contributed to both a* and L; a QTL located at 7.8–20.9 cM on LG5 contributed to both b* and L; and a QTL located at 72.9–92.7 cM on LG7 contributed to all of a*, b* and L. In contrast, a QTL at LG3, a QTL at LG7, and two QTL at LG4 and one QTL at LG6 controlled the parameters a*, b* and L independently.

The contents of seven glucosinolates were scored in leaves of the G-, P- type and F_2_ individuals. In the HPLC-analyses, *para*-hydroxyglucobarbarin and *para*-hydroxyepiglucobarbarin desulfo derivatives coeluted, so only the sum of them was scored (Additional file 2: Table S3) and used in attempts to locate QTL responsible for *para*-hydroxylation. Twelve QTL for five glucosinolates were detected on linkage group 3, 4, 5 and 6, each QTL explaining 2.9–71.3% of the phenotypic variation (PVE) (Fig. 3 and Table 4). One group of overlapping QTL at the upper end of LG4 contributed to 42.3 and 19.3% of the PVE for 2-phenylethylglucosinolate (gluconasturtiin, NAS), 41.6% of the PVE for (2*S*)-2-hydroxy-2-phenylethylglucosinolate (glucobarbarin, BAR) and 71.3% of the PVE for (2*R*)-2-hydroxy-2-phenylethylglucosinolate (epiglucobarbarin, EBAR). Hence, this QTL was the main locus determining levels of all three phenylalanine derived glucosinolates in *B. vulgaris*. However, at the lower end of LG3, overlapping QTL explaining 12.5% PVE of BAR and 6.4% PVE of EBAR were found. Both of these loci displayed strong positive additive effect for BAR and strong negative additive effect for EBAR, indicating a pair of alleles competitively regulating the biosynthesis of this pair of isomers. Two additional QTL for the glucobarbarins (BAR and EBAR) were evident, located at separate loci of LG5: a QTL contributing 9.4% PVE of BAR and a QTL contributing 6.8% PVE of EBAR, which added to the complexity of the control of these isomers. Interestingly, these two QTL of LG5 are co-localized or overlapping with QTL controlling the content of a tryptophan derived glucosinolate (indole glucosinolate), the 4-methoxy derivative 4mIM. This QTL at 11.1–30.9 cM of LG5 contributed 37.2% of PVE of 4mIM, indicating it to be the main QTL for the substituted indole glucosinolate 4mIM. Besides these overlapping loci, an additional QTL in LG4 for 4mIM was identified, accounting for 2.9% of the PVE. Finally, two QTL for the parent indole glucosinolate IM in LG4 and LG5 were identified, accounting for 9.2 and 16.9% of the PVE, respectively.

Overall, it was noticed that 32 of the total of 37 QTL were organized in just seven chromosome regions. In these seven regions, the QTL controlling the leaf size, color and glucosinolates were always overlapping (Fig. 3).

### Glucosinolate profiles in the F_2_ progeny

A considerable variation in glucosinolate profiles was observed in the F_2_ progeny, and was analyzed from a biosynthetic perspective. The glucosinolates were from two biosynthetic families, the tryptophan derived (also known as indole glucosinolates: IM and 4mIM) and the phenylalanine derived (NAS, BAR**,** EBAR and *p*-hydroxy derivatives of BAR and EBAR). Levels of glucosinolates from the two families were only weakly correlated (Additional file 1: Figure S2a). In contrast to the rather similar profile of tryptophan derived glucosinolates in the two parental types, profiles of phenylalanine derived glucosinolates differed markedly, as the G-type was dominated by glucobarbarin (BAR) and the P-type was dominated by the epimer EBAR, differing solely by the stereochemistry of the 2-hydroxyl group. Despite the complex regulation of the phenylalanine derived glucosinolates (the precursor NAS and the epimeric hydroxyl derivatives BAR and EBAR), four apparent main groups of progeny could be distinguished in the glucosinolate profiles (Additional file 1: Figure S2b and c, Additional file 1: Table S3). Two of these were the parental types, the G-type like (labelled *SHO rho*? in Additional file 1: Figure S2b, see discussion) with BAR by far dominating over EBAR and low levels of NAS (*N* = 42), and the P-type like (labelled *sho RHO*? in Additional file 1: Figure S2b), with EBAR dominating and low levels of NAS (*N* = 42) (Accidentally, the two numbers were equal). For scoring, the limit for parental types was set at less than 5% or more than 95% BAR of the combined level of BAR and EBAR, a limit in agreement with visual groups in the progeny (Additional file 1: Figure S2b). A third group with appreciable levels of both BAR and EBAR (labelled *SHO RHO* in Additional file 1: Figure S2b), was common (*N* = 147), while only very few progeny plants were observed in the fourth group (labelled *sho rho* in Additional file 1: Figure S2c) characterized by having high levels of the precursor phenylalanine derived glucosinolate NAS but very low levels of both BAR and EBAR (*N* = 8). Finally, a few F_2_ progeny plants (*N* = 3) combined a moderately high level of NAS with a high level of BAR (Additional file 1: Fig. S2c, Additional file 2: Table S3), and could hence not be unequivocally scored to any category in a simplified two gene model.

Considering the tryptophan derived glucosinolates, there was no correlation between levels of the 4-methoxy derivative 4mIM and the precursor IM (Additional file 1: Figure S2d).

## Discussion

Constructing a high-density linkage map is useful for QTL mapping [33–35], comparative genomics [36] and evolutionary biology research [37], and can even improve assembly of de novo genomic sequences [38]. In the present study, we used RAD-Seq to generate SNP markers and then constructed a high-density linkage map of *B. vulgaris*. A large number of markers were discarded in constructed the linkage map. There are three reasons for the discarded markers. First, the markers absent in > 15% of the total population were discarded. Among 60,287 co-dominant “aa × bb” type SNPs, only 8448 SNPs were present in at least 216 plants (85% of the population) after filtering. Second, markers showing segregation distortion were discarded. Due to the reproductive isolation between the P-type and G-type, the segregation distortion in the F_2_ population is relatively high. Finally, the present marker density is approaching the limit of this technology on an F_2_ population with 255 individuals; adding more markers could introduce more errors and distortions. Compared with the previously published genetic maps [1, 14, 30], the number of individuals in the F_2_ population used for RAD sequencing was 2.3-fold higher, which can improve the detection of lower frequency recombination events. As far as we know, the current map represents the highest density linkage map not only for genus *Barbarea* but also for the crucifer tribe Cardamineae to date. This tribe contains two vegetable and condiment crops, watercress [39] and horseradish [40]. Two genomes have been published from the tribe, for *Cardamine hirsuta* [41] and *Barbarea vulgaris* [1], and the tribe is intensely studied as a case of morphological and molecular defense evolution [15, 17, 20, 30, 42–44].

In this study, we used a 1.94-fold higher number of markers achieving the capture of 722 contigs (50.2 Mb, 30% of the assembly) (Additional file 2: Tables S1 and S2). This is significantly more than previous published maps on which only 431 contigs (38.7 Mb, 23% of the assembly) were mapped [1]. Unfortunately, the published assembly of the *B. vulgaris* genome has up to 7874 contigs with short length (N50 size of 14.3 Kb) and only 62.1% (167.7 Mb) of the estimated genome (270 Mb) was assembled into contigs [1]. Therefore, our present high-density linkage map cannot improve the genome assembly sufficiently using the existing genome sequencing data. With the rapid development of sequencing technologies, continued efforts are needed to improve the genome assembly with third generation sequencing technology.

Because the two maps used different plant populations and different suites of markers, the 150 contigs that anchored to both maps were used as bridges to match the linkage groups (pseudo chromosomes) between each other. Six out of the eight pairs of linkage groups were aligned with strong support. The LG2 and LG8 were supported by only one and three contigs respectively, not as confident as the other six LGs. However, the leaf trichome QTL was located at the LG8 in both of the two maps, suggesting alignment and numbering of the LGs to be reliable. Conflicts of contig anchoring between the two maps were observed within chromosomes and among chromosomes (Additional file 1: Figure S2 and Additional file 2: Table S1), indicating this genome to be highly complex despite its small size. One reason could be that the cruciferous plants have experienced several rounds of genome duplication. The conflicts can be due to the indistinguishable short sequences between subgenomes. Therefore, assembling longer contigs is needed to improve the genome quality.

The G- and P-types of *B. vulgaris* show significant morphological differences with respect to trichome density, and also show qualitative differences in glucosinolate content [5, 12, 13, 45]. Visually apparent differences in leaf color and possibly leaf size has also been observed under both Chinese and Scandinavian conditions but not previously quantified or reported. In this study, a total of 37 QTL were detected for 11 different traits, each QTL explaining 2.9–71.3% of the phenotypic variation. The previous studies on *B. vulgaris* found two QTL for trichome density, one QTL for glucobarbarin (BAR) and one QTL for the epimer epiglucobarbarin (EBAR) [1]. In the present study, these QTL were confirmed, but in addition two new QTL were detected for both BAR and EBAR. The most likely reasons for the more complex genetic model revealed in the present mapping are the enlarged population size (255 individuals of present study vs 111 individuals of previous) and the increased number of markers (1545 vs 796 markers), which improved the QTL detection efficiency [14, 46].

**Fig. 4:**
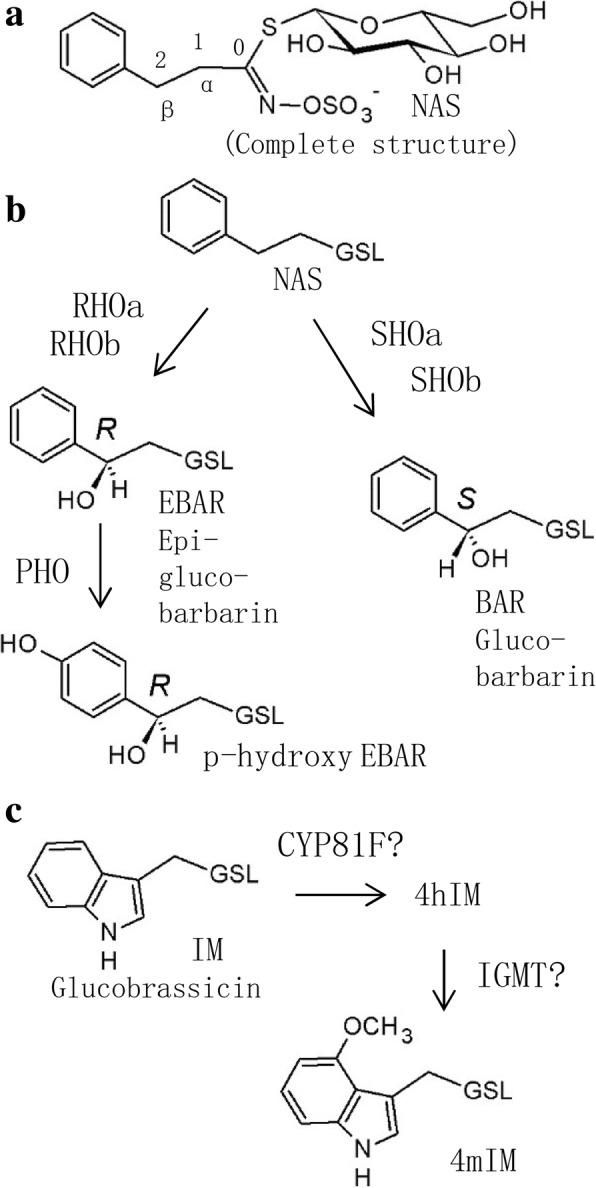
Glucosinolates in *Barbarea vulgaris* leaves as detected in this investigation, with suggested biosynthetic relationships. **a**. General glucosinolate structure, using gluconasturtiin (NAS, 2-phenylethylglucosinolate) as example. b. Biosynthetic relationships of phenylalanine-derived glucosinolates. The general glucosinolate biosynthesis leads to NAS, which is hydroxylated in either of two stereochemical configurations, yielding either BAR (glucobarbarin, (2*S*)-2-hydroxy-2-phenylethylGSL) or EBAR (epiglucobarbarin, (2*R*)-2-hydroxy-2-phenylethylglucosinolate). This hydroxylation was previously anticipated to be carried out by either of two gene products, SHO and RHO. Results of this work suggest involvement of several complementing gene products, provisionally named with suffixes **a** and **b**. A further glucosinolate in the P-type is *p*-hydroxy EBAR (*p*-hydroxyepiglucobarbarin or (2*R*)-2-hydroxy-2-(4-hydroxyphenyl)ethylglucosinolate. **c**. Biosynthetic relationships of tryptophan-derived glucosinolates. The general indole glucosinolate biosynthesis leads to IM (glucobrassicin, indol-3-ylmethylglucosinolate). A homolog of *A. thaliana* CYP81F is expected to lead to the 4-hydroxy derivative 4hIM [38], which is probably taken to the 4-methoxy derivative 4mIM by a homolog of *A. thaliana* IGMT [60]. In all structures except the upper complete structure, the constant glucosinolate backbone is indicated as GSL

In the case of leaf trichomes, our data agree with a previous report of two QTL on LG4 and LG8 [1]. In contrast, our genetic map considerably expands the knowledge of the genetics behind a conspicuous glucosinolate polymorphism in *B. vulgaris*; the polymorphism in stereochemistry of 2-hydroxylation of NAS. The polymorphism is evident in the dominant leaf glucosinolates in the species, which are BAR and EBAR (Fig. 4). Collectively, BAR and EBAR can be called “epimeric glucobarbarins”. From analysis of transcriptome [45] and genome [1] data, and comparison with glucosinolate biosynthesis in *A. thaliana* [47], it is anticipated that NAS, BAR and EBAR are all derived from the amino acid phenylalanine (via homophenylalanine), sharing the general biosynthetic machinery from the amino acid precursor to the simplest of the three glucosinolates, NAS (Fig. 4). From NAS, the biosynthesis is expected to branch, catalyzed by homologs of the *A. thaliana* gene *GS-OH* (glucosinolate hydroxylation). Specifically, a homolog named *SHO* (*S*-*HYDROXYLATION*), which is highly expressed in the G-type but barely expressed in the P-type, was suggested to lead to BAR. In contrast, a homolog named *RHO* (*R*-*HYDROXYLATION*), was suggested to lead to EBAR. This model was supported by the presence of *SHO* in a major QTL for BAR in the *B. vulgaris* genome [1]. In the previous genetic map, only one locus for BAR and one for EBAR was reported, and the loci were unlinked (with the QTL for EBAR in LG4 and the QTL for BAR in LG5) [14].

However, the simple genetic model outlined in previous reports is too simple to account for the complex genetics of epimeric glucobarbarins revealed here. From a model with just two unlinked loci, independent assortment would lead to a 1:1:1:1 ratio of four gene combinations, the parental *SHO rho* and *RHO sho* as well as the recombined *SHO RHO* and *sho rho*. From the F_1_ phenotype of approximately equal levels of BAR and EBAR [45], the major genes are known to be codominant, meaning that these gene combinations would each be predictable from the phenotype, with *SHO RHO* having relatively high levels of BAR and EBAR and *sho rho* having relatively low levels of BAR and EBAR, combined with high levels of NAS because of the still ongoing general glucosinolate biosynthesis leading to accumulation of the precursor of BAR and EBAR. The observation of a high proportion of recombinant progeny was in agreement with several genetic models, however, the low frequency (5.6% or 7.7% depending on interpretation of glucosinolate profiles) of apparent *sho rho* progeny plants, with low levels of both BAR and EBAR combined with a high level of NAS, was in disagreement with a model of two unlinked genetic loci of *SHO*/*sho* and *RHO*/*rho*. The same unexpected deficiency in offspring dominated by NAS had also been reported from the first *B. vulgaris* map but not discussed [14].

The present discovery of multiple QTL for the epimeric glucobarbarins (BAR and EBAR), in combination with the recently published genome, provides a potential explanation of these consistent recombination proportions. Investigation of the genome revealed three different genes in the G-type with sequence similarity to *GS-OH*, named *GS-OH-like 1*, *− 2* and *− 3*. Of these, *GS-OH-like 1* was found identical to *SHO* and had an allele with very low expression in the P-type (*sho*) [1]. The number of homologs in the P-type was not determined with certainty. Assuming several *GS-OH* like genes in each type of *B. vulgaris*, the dominance of progeny plants high in both BAR and EBAR, and the scarcity of progeny plants low in both epimeric glucobarbarins and high in NAS, can be explained. Obvious fine structure in the group observations attributed to SHO RHO (Additional file 1: Figure S2b) is likewise in accordance with the suggested complex genetic model. In this biosynthetic/genetic model (Fig. 4), this suggestion is illustrated with additional homologs of *SHO* and/or *RHO*, provisionally named with suffixes a and b (etc.). However, despite the added complexity from additional loci for 2-hydroxylation, we suggest that some *sho rho* progeny plants were correctly identified from glucosinolate profiles (Additional file 1: Figure S2c), while some progeny plants of more complex recombinant genotypes (e.g. of the type *sho a SHO b* / - -) may have appeared like the parental types or *SHO RHO* types in Additional file 1: Figure S2b. A combination of *sho rho* with compensatory “*SHOb*” genes with intermediate penetrance could lead to the three atypical plants with appreciable levels of both NAS and BAR, as well as numerous progeny plants with slightly above normal levels of NAS (Additional file 1: Figure S2c). According to this hypothesis, the QTL for BAR and EBAR in LG3, LG4 (and possibly LG5, see below) would be due to homologs of *GS-OH* or any other gene family encoding hydroxylating enzymes. Indeed, it is currently not certain whether β-hydroxylation of glucosinolates generally occurs via GS-OH enzymes, and whether single alleles of *GS-OH* in *Arabidopsis* and *Brassica* are a result of convergent evolution [48]. Hydroxylation of glucosinolate side chains is of huge economic importance, being critical for the nutritional value of many vegetables and industrial crops [49], and a better understanding of its biochemistry can influence breeding in many species.

Considering indole glucosinolates (IM and 4mIM, Fig. 4), the genetics and biochemistry in *A. thaliana* is increasingly well understood [50, 51]. No qualitative or quantitative difference of 4mIM between the two *B. vulgaris* types was realized before this investigation [13]. Assuming similarity to *A. thaliana* [45], any specific QTL for 4mIM could represent the first hydroxylation step (*CYP81F*) or the second methylation step (a homolog of *IGMT*) or a regulatory locus (Fig. 4c). However, because of possible overlap of QTL for 4mIM, BAR and EBAR on LG5 and of IM and 4IM on LG4, the loci could also represent more general control of glucosinolate biosynthesis, so speculation on the nature of these possibly overlapping loci would seem premature.

A further glucosinolate in the P-type is the *para*-hydroxyl derivative of EBAR (Fig. 4), which gives rise to a unique type of hydrolysis product [12]. In F_1_ progeny and other plants with high levels of BAR, EBAR and even NAS, *para*-hydroxyl derivatives of the dominating glucosinolates accumulate, suggesting a gene for *para*-hydroxylation of phenylalanine derived glucosinolates in general. To our surprise, no QTL was detected for *p*-hydroxy derivatives of epimeric glucobarbarins (Fig. 4). Lack of detection of this expected QTL could be due to the very low levels of *p*-hydroxyls observed in our progeny plants, which may lead to analytical inaccuracy due to possible peak overlap with impurities.

The content of NAS in the crop watercress is well established. More recently, it was reported that many commercial accessions from the USA also contain epimeric glucobarbarins, which are of unknown nutritional and toxicological importance [52]. Molecular data for watercress are accumulating [52–55], but high quality genetic maps are yet lacking. As *Barbarea* and watercress are related [40] and share the same basic chromosome number (*n* = 8), the present high-density map may be of relevance also for watercress breeding.

The observed glucosinolate profiles mirror characteristic variation known from within-plant differences and from natural populations. While relative levels of EBAR and BAR are highly skewed in leaves, they are typically less skewed in roots [13] and in seeds of some accession [12], all in accordance with a model of several homologs that could vary in tissue-specific expression. In nature, the G-type and P-type of *B. vulgaris* occupy separate geographic areas, with a meeting zone somewhere in Eastern/Central Europe [4]. Indeed, near that meeting zone apparent hybrid populations were detected [13]. One such hybrid population from Finland showed F_1_ like glucosinolate profiles, while a population from Slovenia contained a high proportion of individuals dominated by NAS (“NAS-forms”). In another population, from the Caucasus, individuals with high proportions of both NAS and EBAR (and further peculiarities) were observed, reminiscent of the rare phenotypes high in both NAS and BAR observed in F_2_ progeny. The present work provides an explanation of how these aberrant phenotypes could have originated from P × G type hybridization and genetic recombination, and confirms creation of such new glucosinolate profiles by controlled hybridisation. Similar aberrant phenotypes have been reported from the Netherlands [56], far from the meeting zone of the types, and were found to correlate with marked ecological effects [57], although a causal relationship has recently been questioned [58].

To the best of our knowledge, this is the first report of QTL for leaf size, leaf color, gluconasturtiin (NAS) and indole glucosinolates of *B. vulgaris* rosette leaves. In addition, our analysis provides further details on the genetic regulation of epimeric glucobarbarins, the dominating glucosinolates in the parental types. Interestingly, most of the QTL were overlapping and enriched at seven chromosome regions. For example, the QTL regions for maximum leaf length and width, L, a* and b* are overlapped on LG3, LG5, and LG7. This can be explained by assuming that L, a* and b* are representations of components of photosynthetic pigments which directly controls the photosynthesis and therefore affect leaf size. However, we had not anticipated the QTL for glucosinolates to be co-localized with those of leaf size and color components. This finding will impel us to recognize the relationship between the secondary metabolism and plant development. However, a role of glucosinolates in plant growth regulation is currently being unravelled in *A. thaliana* [59], so an effect of glucosinolate biosynthetic genes on general plant growth parameters is not unlikely. Fine-mapping of these QTL by enlarging the mapping population and finally cloning of the genes using improved genome sequences would be worth doing in the future.

## Conclusions

In conclusion, the present study reports the highest density linkage map to date for the crucifer tribe Cardamineae. On the basis of the present high-density genetic map, an improvement of genome contigs was anchored compared to a previously published map. Besides, 37 QTL were detected for eleven traits of glucosinolates, leaf size and color, each QTL explaining 2.9–71.3% of the phenotype variation. The QTL obtained in this study will be useful for further understanding the molecular genetic basis of these traits, many of which are shared in the related crop watercress.

## Methods

### Plant material and cultivation

Seeds of *B. vulgaris* accessions B4 (P-type, NGB23547) and B44 (G-type, NGB31789) and F_1_ hybrid were obtained as previously reported [45]. Both accessions are available from www.nordgen.org/sesto and registered under the indicated NGB numbers. The 255 F_2_ individuals in the mapping population were generated from self-pollination of a single F_1_ plant. All plants were grown in the Institute of Vegetables and Flowers, Chinese Academy of Agricultural Sciences, China, an area having freezing winters and hot summers. Seeds of the F_2_ generation were surface sterilized in 1% NaClO and sown in 9 × 9 cm pots filled with a mixture of peat and vermiculite (V:V = 2:1) on March 5, 2015. Plants were kept in a growth chamber until transplant atconditions as in our previous report [45]. The seedlings were transplanted to a plastic tunnel with a row spacing of 50 cm and a plant spacing of 30 cm on April 30. Plants were given fertilizer and water as needed.

### Genomic DNA extraction

Young leaves of F_2_ plants and their parents were collected, freeze-dried and ground to a fine powder. Genomic DNA was extracted from 100 mg leaf sample for each plant using the CTAB method [60]. The quality of the DNA was assessed by 1% agarose gel electrophoresis and Nano Photometer® (TIMPLEN,CA, USA), and the concentrations were measured by a Qubit® DNA Assay Kit in a Qubit® 2.0 Fluorimeter (Life Technologies, CA, USA).

### RAD-Seq library construction and Illumina sequencing

The RAD-Seq libraries were constructed according to the protocol of Baird et al. [33]. The restriction enzyme *EcoR* I was used to digest genomic DNA and P1 adapter (contains forward amplification and Illumina sequencing primer sites, as well as a nucleotide barcode 6 bp long for sample identification) was ligated to the fragment’s compatible ends. The adapter-ligated fragments were subsequently polled, randomly sheared, and size-selected. Then, the fragments were ligated to a P2 adapter, a Y adapter that has divergent ends. PCR amplification was conducted and DNA fragments spanning 200–600 bp were isolated on a 1% agarose gel and purified for library construction. The qualities of the libraries were ensured, and then paired-end (150 bp) sequencing was carried out on an Illumina HiSeq platform.

### RAD-Seq sequence analyses and SNP markers development

Raw sequence reads of ‘.fastq’ format were segregated by the barcode assigned to each sample and filtered to remove low-quality sequences and trimmed adaptor. Reads with ambiguous ‘N’ nucleotides exceeding 10% of read length were eliminated. Sequences in which more than 50% of the nucleotides had a Phred score below 5 were also considered low quality and eliminated. The trimmed reads of G-type and P-type *B. vulgaris* were clustered based on sequence similarity using the cd-hit-test with parameters of less than 3 nucleotides mismatch [61]. Clusters with read depth very low (less than 10) or very high (more than 400) were excluded.

The paired-end reads of each cluster passing the above tests were then subjected to de novo assembly to generate contigs using the VelvetOptimizer [62] with parameters of “VelvetOptimiser.pl -s 23 -e 31 -x 4”. Contigs less than 125 bp were discarded. The remainder of the contigs were used as reference genome for other analysis. Sequencing reads were aligned to the reference genome using BWA software (settings: mem -t 4 -k 32 -M). Alignment files were converted to BAM files using SAM tools software [63]. Variant allele calling was performed for each individual by using the mpileup function in SAM tools (settings: mpileup -q 1 -C 50 -S -D -m 2 -F 0.002) [64]. Polymorphic contigs in the G-type and P-type *B. vulgaris* were identified by the presence of at least 10 reads in one type and none in the other [65].

Polymorphic reads were mapped to the P-type (reference sequence) first to identify perfect matches as RAD marker sites and then searched for SNPs [33]. The types of SNPs in individual F_2_ progeny were identified by the presence of at least 4 but no more than 1000 reads.

### Linkage map construction

RAD markers genotyped with more than 15% missing data in the F_2_ progeny were removed. The marker segregation frequencies were determined using chi-squared analysis to test the goodness of fit to the expected segregation ratio of 1:2:1. Markers showing significant segregation distortion at *P* < 0.001 were excluded in the present study [66]. JoinMap 4.0 was used for recombination calculation and the genetic map construction [67]. After having removed the similar sites (similarity > 0.95), the remaining markers were divided into eight groups and the marker order of each linkage group was ordered using the regression mapping algorithm of JoinMap 4.0. The severely unlinked markers were discarded and the MapChart program was used to visualize exported maps [68].

### Alignment of linkage groups with the contigs and synteny block with previous genome sequencing

To keep the correspondence of our present linkage groups with the eight *B. vulgaris* chromosomes, we aligned our high density genetic map with the contigs (http://plen.ku.dk/Barbarea) generated by genome sequencing of G-type *B. vulgaris* using a Perl script. Furthermore, the visualized map of corresponding markers of the two high-density linkage maps were generated based on the commonly harbored contigs.

### Phenotyping and QTL detection

Forty-one days after transplanting (June 10, 2015), the trichome density of the youngest and four additional randomly selected true leaves was scored on a 0–4 scale [4] with a little modification: 0 = no trichomes; 1 = few, occasional trichomes on leaf margin; 2 = several trichomes on leaf margin and veins, but none on lamina; 3 = many trichomes, also on lamina, but significantly less than P-type plants; 4 = many trichome, also on lamina, similar to or surpassing the P-type. For each of the F_2_ individuals, the largest leaf was selected and pressed flat on a surface to measure the end-lobe by a ruler, thereby determining the maximum leaf length (cm) and maximum leaf width (cm) at 30 days after transplanting. The leaf colors were observed by a CR-400 color difference meter (Konica Minolta, Shanghai, China) and three randomly selected true leaves per plant were measured. The leaf color was presented according to CIE 1976 Lab standards [69] by L (lightness), a* (redness and greenness) and b* (yellowness and blueness) with a standard D65 light source. For glucosinolate analysis, four true leaves (including petiole and side lobes) among those of intermediate developmental stage of each F_2_ individual were randomly sampled on July 20, 2015. The leaf samples were freeze-dried at − 80 °C and then ground into a fine powder. Two hundred mg of leaf powder was used for glucosinolate analysis by HPLC of desulfated derivatives as in our previous report [45].

Putative QTL location was determined by the composite interval mapping (CIM) method using winQTLcart [70]. The phenotypic variance explained (PVE), as well as additive genetic effects and intervals on maps by each QTL were obtained. The LOD score threshold (α = 0.05) for determining the presence of a QTL was calculated using the permutation test (1000 replications) [71].

## Additional files

Additional file 1: **Figure S1.** The number and coverage of RAD-tags for G-type, P-type and each F_2_ individual of *Barbarea vulgaris*. The x-axis indicates the plant accession including G-type, P-type and each of the F_2_ individuals; the y-axis indicates the number of RAD-tags (a) and the coverage (b). **Figure S2.** Characteristics of the glucosinolate profiles in F_2_ offspring from a cross between P-type and G-type *B. vulgaris*. a. Glucosinolates from each of the two biosynthetic families, tryptophan (Trp) derived and phenylalanine (Phe) derived, showed little correlation. b. Three large, rather well defined groups of progeny plants could be recognized from the balance of the glucosinolate epimers BAR and EBAR. For the inserted hypothetic genotypes, see Discussion. The question marks indicate that the genotypes may be overly simplified, due to additional genes influencing the glucosinolate profile. In addition, a further fine structure in the group *SHO RHO* is evident, with one group (the upper) relatively higher in EBAR than the other. c. A plot of NAS levels as a function of the sum of its two hydroxyl derivatives BAR and EBAR revealed a small deviating group of ‘NAS-form’ progeny plants, characterized by very low levels of both epimeric glucobarbarins (BAR and EBAR), resulting in accumulation of the apparent biosynthetic precursor, NAS. Reasons for the very low number of apparent *sho rho* plants are discussed in Discussion. d. Levels of the 4-substituted indole glucosinolate 4mIM were essentially not correlated with the precursor IM, suggesting the substitution to be specifically regulated, in agreement with identification of a major QTL for 4mIM (ZIP 238 kb)

Additional file 2: **Table S1.** Anchoring of sequenced contigs of G-type *Barbarea vulgaris* to the eight linkage groups. **Table S2.** Comparison of anchored contig information between this and the best previous *Barbarea vulgaris* genome assembly. **Table S3.** The trichome density, color, maximum leaf length and width, and glucosinolate content in the leaves of each individual of the F_2_ population of *Barbarea vulgaris (ZIP 1442 kb)*

## Abbreviations

4mIM: 4-methoxy-3-indolylmethylglucosinolate (4-methoxyglucobrassicin)
a*: Redness and greenness
AFLP: Amplified fragment length polymorphism
b*: Yellowness and blueness
BAR: (*2S*)-2-hydroxy-2-phenylethylglucosinolate (glucobarbarin)
CIM: Composite interval mapping
CTAB: Cetyl trimethyl ammonium bromide
EBAR: (*2R*)-2-hydroxy-2-phenylethylglucosinolate (epiglucobarbarin)
GS-OH: Glucosinolate hydroxylation
G-type: Glabrous type
HPLC: High performance liquid chromatograph
IM: 3-indolylmethylglucosinolate (glucobrassicin)
L: Lightness
LG: Linkage group
MLL: Maximum leaf length
MLW: Maximum leaf width
NAS: 2-phenylethylglucosinolate (gluconasturtiin)
P-type: Pubescent type
PVE: Phenotypic variation
QTL: Quantitative trait locus
RAD-Seq: Restriction site-associated DNA sequencing
RHO: R-HYDROXYLATION
SHO: S-HYDROXYLATION
SNP: Single nucleotide polymorphism
SSR: Simple sequence repeat
