# Supplementary figures and images for: A high-density genetic map and QTL mapping of leaf traits and glucosinolates in Barbarea vulgaris
Source: BMC Genomics. 2019 May 14;20:371. doi: 10.1186/s12864-019-5769-z (PMC6518621; doi:10.1186/s12864-019-5769-z)

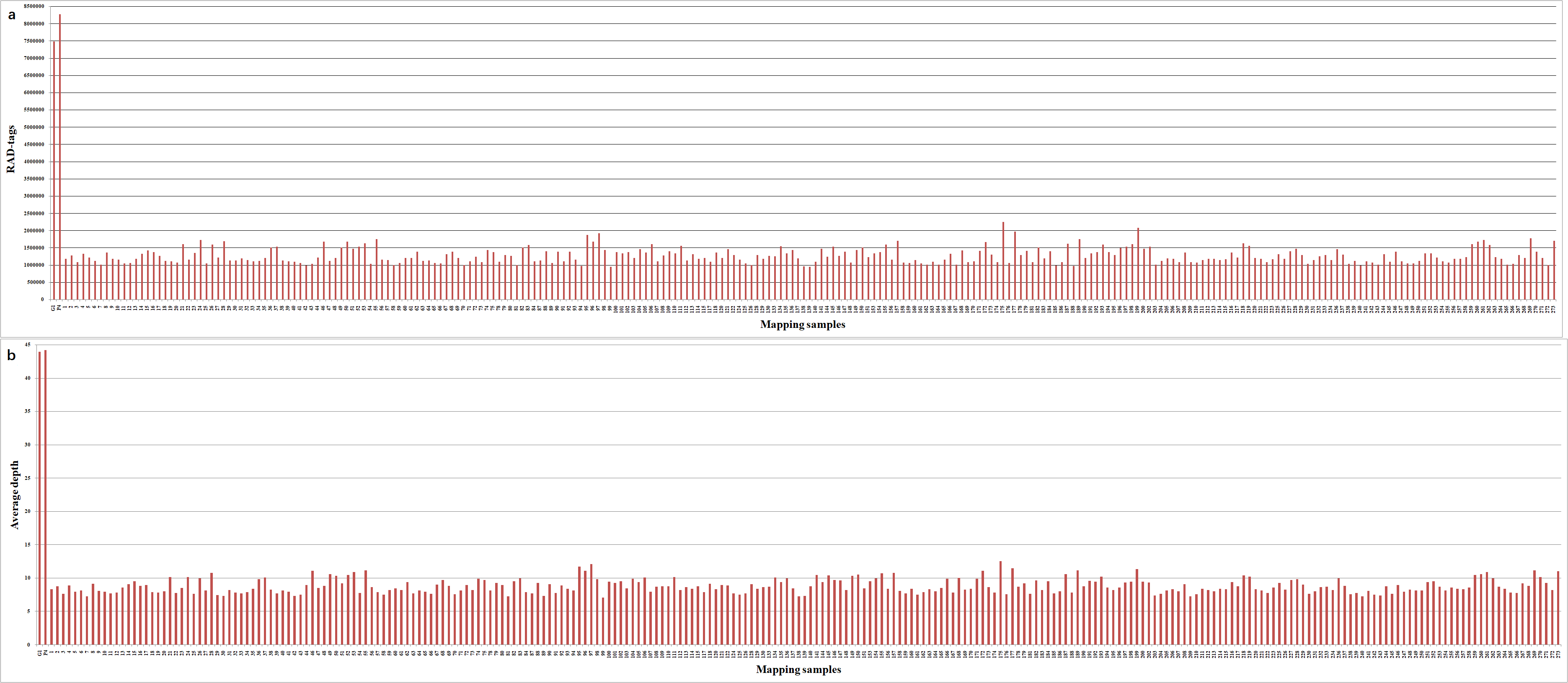

Supplement: Supplementary file 1 — Figure S1. The number and coverage of RAD-tags for G-type, P-type and each F2 individual of Barbarea vulgaris. The x-axis indicates the plant accession including G-type, P-type and each of the F2 individuals; the y-axis indicates the number of RAD-tags (a) and the coverage (b). Figure S2. Characteristics of the glucosinolate profiles in F2 offspring from a cross between P-type and G-type B. vulgaris. a. Glucosinolates from each of the two biosynthetic families, tryptophan (Trp) derived and phenylalanine (Phe) derived, showed little correlation. b. Three large, rather well defined groups of progeny plants could be recognized from the balance of the glucosinolate epimers BAR and EBAR. For the inserted hypothetic genotypes, see Discussion. The question marks indicate that the genotypes may be overly simplified, due to additional genes influencing the glucosinolate profile. In addition, a further fine structure in the group SHO RHO is evident, with one group (the upper) relatively higher in EBAR than the other. c. A plot of NAS levels as a function of the sum of its two hydroxyl derivatives BAR and EBAR revealed a small deviating group of ‘NAS-form’ progeny plants, characterized by very low levels of both epimeric glucobarbarins (BAR and EBAR), resulting in accumulation of the apparent biosynthetic precursor, NAS. Reasons for the very low number of apparent sho rho plants are discussed in Discussion. d. Levels of the 4-substituted indole glucosinolate 4mIM were essentially not correlated with the precursor IM, suggesting the substitution to be specifically regulated, in agreement with identification of a major QTL for 4mIM (ZIP 238 kb) [file 12864_2019_5769_MOESM1_ESM.zip › Fig. S1.png]

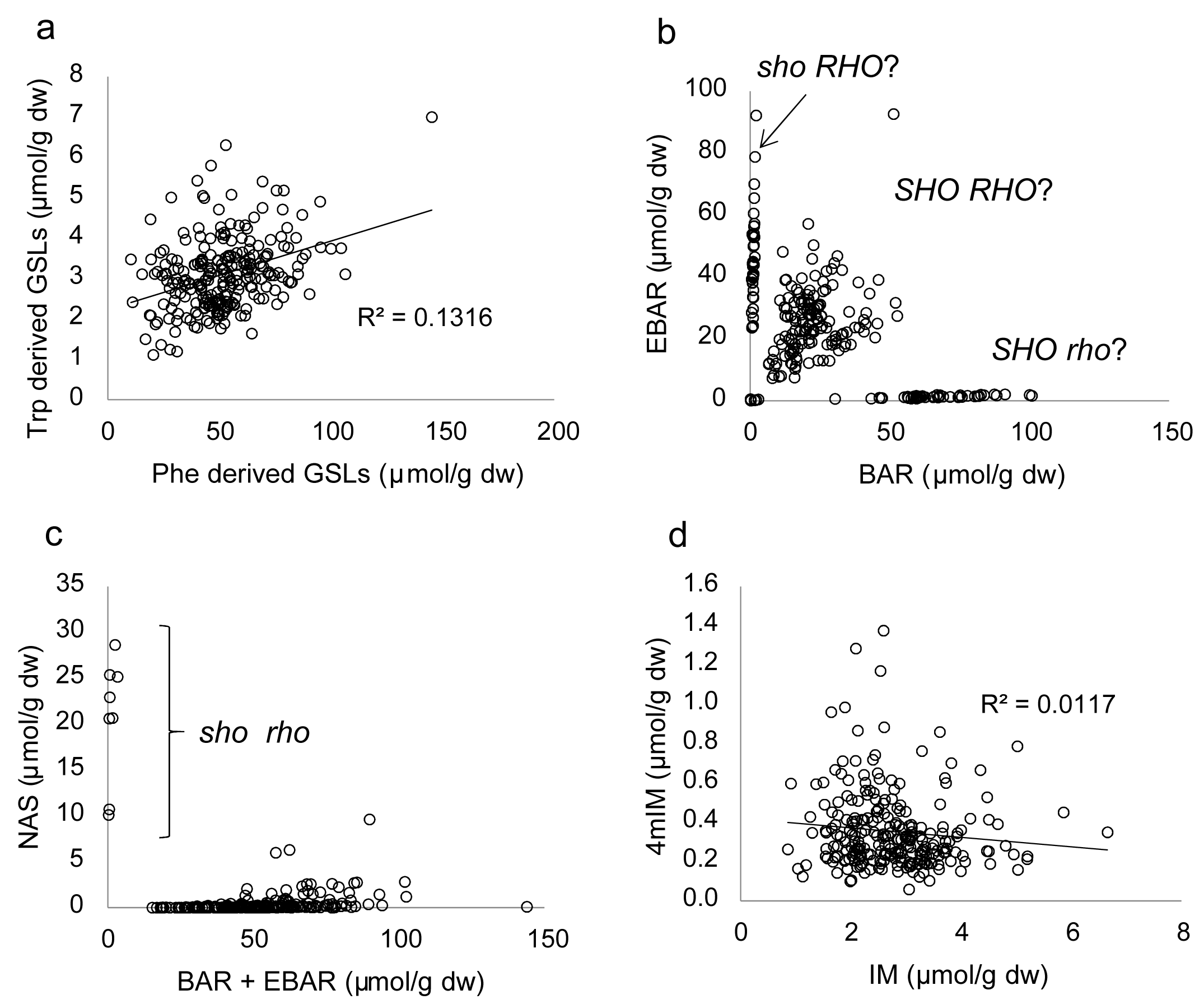

Supplement: Supplementary file 1 — Figure S1. The number and coverage of RAD-tags for G-type, P-type and each F2 individual of Barbarea vulgaris. The x-axis indicates the plant accession including G-type, P-type and each of the F2 individuals; the y-axis indicates the number of RAD-tags (a) and the coverage (b). Figure S2. Characteristics of the glucosinolate profiles in F2 offspring from a cross between P-type and G-type B. vulgaris. a. Glucosinolates from each of the two biosynthetic families, tryptophan (Trp) derived and phenylalanine (Phe) derived, showed little correlation. b. Three large, rather well defined groups of progeny plants could be recognized from the balance of the glucosinolate epimers BAR and EBAR. For the inserted hypothetic genotypes, see Discussion. The question marks indicate that the genotypes may be overly simplified, due to additional genes influencing the glucosinolate profile. In addition, a further fine structure in the group SHO RHO is evident, with one group (the upper) relatively higher in EBAR than the other. c. A plot of NAS levels as a function of the sum of its two hydroxyl derivatives BAR and EBAR revealed a small deviating group of ‘NAS-form’ progeny plants, characterized by very low levels of both epimeric glucobarbarins (BAR and EBAR), resulting in accumulation of the apparent biosynthetic precursor, NAS. Reasons for the very low number of apparent sho rho plants are discussed in Discussion. d. Levels of the 4-substituted indole glucosinolate 4mIM were essentially not correlated with the precursor IM, suggesting the substitution to be specifically regulated, in agreement with identification of a major QTL for 4mIM (ZIP 238 kb) [file 12864_2019_5769_MOESM1_ESM.zip › Fig. S2.tif]
