# Supplementary material for: A high-density genetic map and QTL mapping of leaf traits and glucosinolates in Barbarea vulgaris
Source: BMC Genomics. 2019 May 14;20:371. doi: 10.1186/s12864-019-5769-z (PMC6518621; doi:10.1186/s12864-019-5769-z)
Supplement: Supplementary file 2 — Table S1. Anchoring of sequenced contigs of G-type Barbarea vulgaris to the eight linkage groups. Table S2. Comparison of anchored contig information between this and the best previous Barbarea vulgaris genome assembly. Table S3. The trichome density, color, maximum leaf length and width, and glucosinolate content in the leaves of each individual of the F2 population of Barbarea vulgaris (ZIP 1442 kb) [file 12864_2019_5769_MOESM2_ESM.zip › Table S2.docx]

**Table S2 Comparison of anchored contig information between this and the best previous *Barbarea vulgaris* genome assembly**

| Number of anchored contigs | Size of anchored contigs (Mb) | Anchored contigs in previous genome | Size of anchored contigs in previous genome (Mb) | Common contigs | Size of common contigs (Mb) | Newly anchored contigs | Size of newly anchored contigs (Mb) |
| --- | --- | --- | --- | --- | --- | --- | --- |
| 722 | 50.2 | 431 | 38.7 | 150 | 19.0 | 572 | 31.2 |
